# Cytokines as Mediators of Chemotherapy-Associated Cognitive Changes: Current Evidence, Limitations and Directions for Future Research

**DOI:** 10.1371/journal.pone.0081234

**Published:** 2013-12-05

**Authors:** Yin Ting Cheung, Si Rong Lim, Han Kiat Ho, Alexandre Chan

**Affiliations:** 1 Department of Pharmacy, National University of Singapore, Singapore, Singapore; 2 Department of Pharmacy, National Cancer Centre Singapore, Singapore, Singapore; "Mario Negri" Institute for Pharmacological Research, Italy

## Abstract

**Objectives:**

While various clinical and pharmacological determinants for chemotherapy-associated cognitive impairment have been identified, conflicting evidence suggests that cytokines might play an intermediary role. The objective of this systematic review was to evaluate the current evidence pertaining to the associations among chemotherapy, cytokines induction and cognitive impairment in cancer patients.

**Methods:**

A literature search with PubMed and SciVerse Scopus was conducted in March 2013 to gather relevant articles and abstracts that fulfilled the inclusion and exclusion criteria. This review included studies that had performed objective and/or subjective cognitive assessments and cytokine measurements on defined populations of cancer patients who received chemotherapy.

**Results:**

High methodological heterogeneity existed among the selected studies which differed in cancer populations, subject characteristics, cognitive endpoints, types of cytokines tested and their measurement methods. Weak to moderate correlations were observed between IL-1β, IL-6, TNF-α levels, and different degrees of cognitive impairment. Different types of chemotherapy treatments might lead to varying presentations and severities of cytokine-induced cognitive impairment. Notably, the time concordance between the onset of cytokine induction and occurrence of cognitive impairment was not well elucidated. A number of confounding factors was identified to interfere with the expression levels of cytokines; these confounders included subjects' cancer types, ages, genders, genetics and psychosocial characteristics such as anxiety, depression and fatigue.

**Conclusion:**

Although existing studies observed cognitive impairment and cytokine dysregulation in patients who receive chemotherapy, our results suggest that the intermediary role of cytokines in post-chemotherapy cognitive impairment is still controversial and requires further evaluation. A list of methodological recommendations is proposed to harmonize future studies of this subject matter.

## Introduction

Numerous recent studies have been conducted on cognitive changes in breast cancer patients[1]–[3]. These cancer survivors commonly experience a subtle yet notable change in cognitive function after receiving chemotherapy, a phenomenon termed “chemobrain” or “chemofog”. The reported prevalence of cognitive impairment varies widely from 6 to 75%, with patients experiencing moderate to severe levels of impairment across studies[3], [4]. Cognitive impairment includes deficits in various cognitive domains, such as impaired memory, alertness or attention, learning, processing speed and executive functioning. As cognitive changes have an adverse effect on cancer patients' daily functioning and quality of life[2], [5]–[7], much recent research has attempted to ascertain the determinants and mechanisms that contribute to this “chemobrain” phenomenon.

Multiple factors have been postulated as determinants of cognitive changes in cancer patients, including demographical, physiological, psychological, pathological and pharmacological determinants[4], [8], [9]. Notably, recent experimental studies have suggested that pro-inflammatory cytokines may be mediators of chemotherapy-associated cognitive changes[10]–[17]. Cytokine induction in the central nervous system has been suggested to mediate "sickness behavior" in patients with severe infection or cancer, together with the adverse neuropsychiatric effects of treatment with interferon and interleukins[13]. It has been widely acknowledged that cytokines and inflammatory markers may give rise to a cluster of cancer-related symptoms including fatigue, depression and stress, which are also associated with cognitive changes[10]–[30]. In summary, cytokines play a dominant role in the neuroimmunoendocrine processes-induced by cancer cells and cytotoxic chemotherapy.

**Figure 1:**
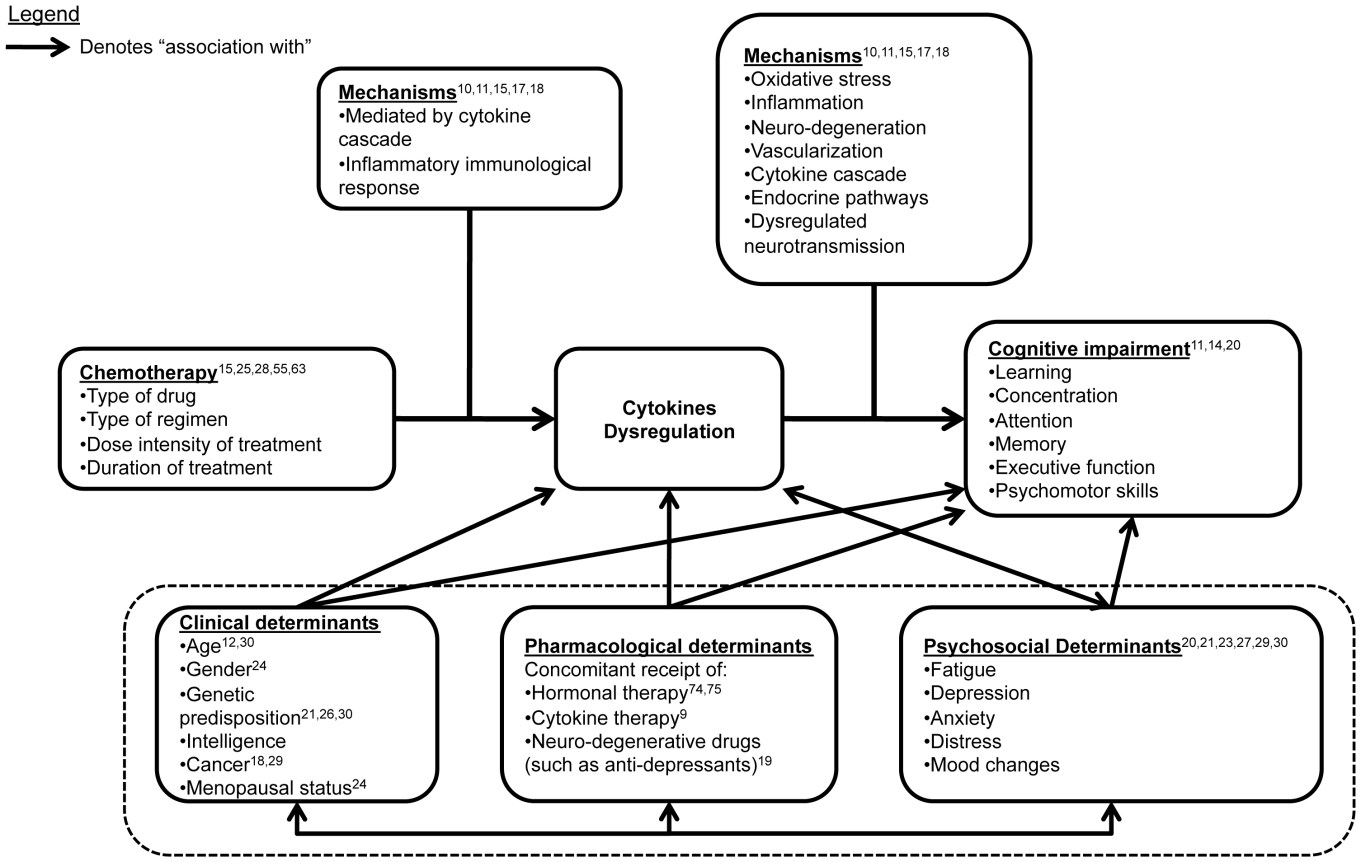
Role of cytokines and other determinants in chemotherapy-induced cognitive impairment. Multiple factors have been postulated as determinants of cognitive changes in cancer patients, including demographical, physiological, psychological, pathological and pharmacological determinants. Recent experimental studies have suggested that pro-inflammatory cytokines may be mediators of chemotherapy-associated cognitive changes.

Chemotherapeutic agents, which are mostly unable to cross the blood-brain-barrier (BBB) due to its molecular size, can cause toxicity to the brain indirectly via the pro-inflammatory cytokine pathways. Cytokines can then penetrate the BBB readily by active transport and through circumventricular regions in the brain[30], [31]. They bind to the endothelial receptors in the brain vasculature to stimulate the release of other inflammatory mediators, such as cell adhesion molecules, chemokines, nitric oxide and prostaglandins, that impede the integrity of the BBB[32], [33]. In the brain, cytokines elicit local inflammation through oxidative and nitrosative processes, especially in the hippocampus and regions of the brain with abundant cytokine receptors[34]–[40]. For example, animal studies have suggested that doxorubicin could increase the level of circulating TNF-α that penetrates the BBB to cause the induction of nitric oxide synthase leading to overproduction of reactive nitric species and reactive oxygen species that act on brain mitochondria to cause oxidative stress[10]. These reactions would ultimately lead to the clinical presentations of cognitive dysfunction[15], [16], [41]–[44]. Figure 1 provides a brief summary of how cytokines may function as a mechanistic mediator in chemotherapy-associated cognitive changes.

To gain a better understanding of the role that cytokines play in the “chemobrain” phenomenon, it is necessary to evaluate the sequence of events involving chemotherapy and the cytokine-based immune response that affects cognitive functioning in cancer patients. Thus far, there is a paucity of studies specifically evaluating the relationship between chemotherapy, cytokine induction and cognitive changes. This review is designed to investigate the possible mediatory role that cytokines play in the neurocognitive sequelae of chemotherapy, and to identify potential confounding factors that may affect the accurate assessment of cytokines as biological determinants of chemotherapy-induced cognitive impairment. The review findings are used to propose a set of methodological guidelines for studies involving this subject matter.

## Methods

A literature search was conducted using PubMed and SciVerse Scopus in March 2013. Only articles in English published before March 2013 were reviewed. The search was carried out using the following combination of keywords: “cancer,” “chemotherapy,” “cytokines,” “chemokines,” “cognitive,” “neuropsychological,” “neurotoxicities,” “chemobrain,” “chemofog,” “tumor necrosis factor or TNF,” “interferon or IFN,” “interleukin or IL,” “colony-stimulating factors or CSF,” “growth factors,” “memory,” “concentration,” “processing speed,” “learning,” and “alertness.” The Medical Subject Headings (MESH) application in PubMed was also utilized. The review included original reports and abstracts from oncology meetings, such as the American Society of Clinical Oncology (ASCO), the European Society for Medical Oncology (ESMO), the San Antonio Breast Cancer Symposium (SABCS), the Multinational Association of Supportive Care in Cancer (MASCC) and the International Cognition and Cancer Task Force (ICCTF). Additional information was obtained from virtual and poster presentations for abstracts presented at oncology meetings.

The reviewed studies met the following inclusion criteria: (1) clinical studies conducted on defined cancer populations; (2) included the administration of cytotoxic chemotherapy drugs or regimens; (3) involved the quantitative measurement of a specific panel of cytokines, such as interleukins (IL), interferon (IFN), tumor necrosis factor (TNF), soluble TNF receptor (sTNF-R), growth factors and colony-stimulating factors (CSF); and (4) included the objective and/or subjective assessment of overall cognition or at least one cognitive domain.

Studies were excluded if they were meta-analyses, reviews, commentaries or case studies; if they focused on peripheral neurotoxicity or brain malignancy-related cognitive impairment; or if cytokine and/or cognitive measurements were omitted. The corresponding authors of the included articles were contacted when additional information on the study was needed. Data extraction and summary of study results were conducted by the investigators independently, and any disparities in the findings were reconciled.

## Results and Discussion

### Overview of Literature Search

**Figure 2:**
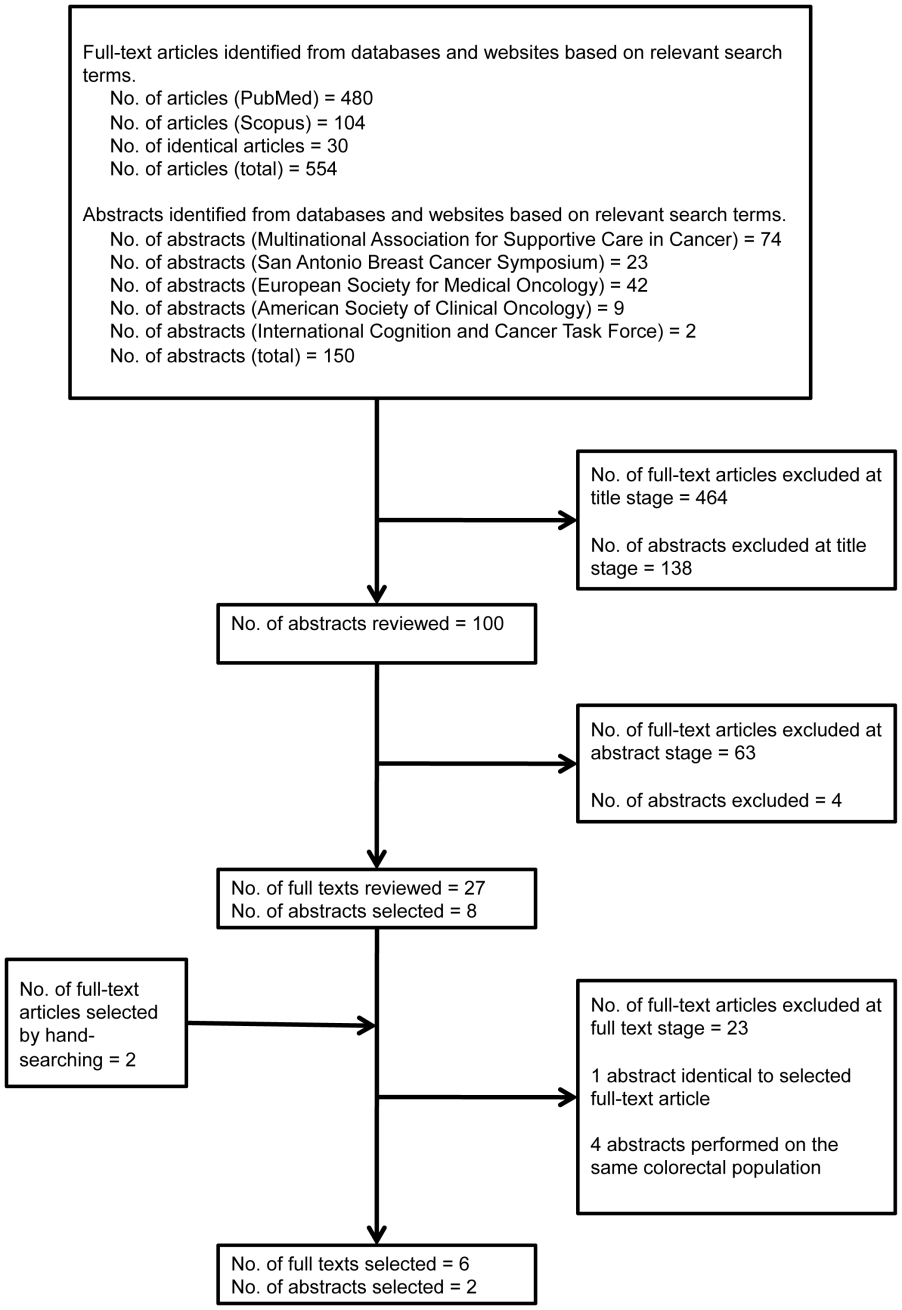
Results of Literature Search.

The results of the literature search are depicted in Figure 2. Thirteen selected articles fulfilled the inclusion criteria[22], [41], [45]–[55], of which six articles were full texts and seven articles were abstracts presented at oncology meetings. Four abstracts [45]–[48] were found to involve the same colorectal cancer populations at different times; therefore, the abstract with either the latest or the most comprehensive set of data was utilized in the review. One abstract[54] was identical to a full text article[53].

### Heterogeneity of Studies

Despite fulfilling the inclusion and exclusion criteria, the objectives of most of the included studies were not fully aligned with the purpose of determining the relationship between chemotherapy, cytokine levels and cognitive impairment. Some studies did not investigate the association between post-chemotherapy cognitive changes and cytokine dysregulation. There was a high level of heterogeneity among the selected studies. These inconsistencies included variations in patient characteristics, types of chemotherapy, types of cytokines measured, methods of measuring cytokines and types of cognitive assessments. Heterogeneity existed because cognitive function was not the primary endpoint in some of the selected studies. For example, two studies[22], [50] were designed primarily to investigate the effects of chemotherapy and cytokine levels on patients' quality of life and fatigue.

The included studies were conducted on a variety of cancer populations, including breast cancer[41], [49], [53], [55], colorectal cancer[45], head and neck cancer[51], lymphoma[50] and acute myelogenous leukemia[22]. Hence, the type, dose intensity, route of administration and duration of chemotherapy treatment varied across the studies. The common chemotherapy drugs of interest were doxorubicin, cyclophosphamide, methotrexate, irinotecan and oxaliplatin. Examples of chemotherapy regimens that were administered to patients included CAF/AC[55], CMF[55], BEAC[50] and CHOP[50] (see Table 1).

**Table 1:** Summary of methods and results from selected studies (Studies are listed in alphabetical order).

Author	Study design	Subjects		Chemotherapy regimens b	Cytokines tested c		Neuropsychological assessments d			Results and study conclusion e	
		Chemotherapy receiving	Non-chemotherapy receiving		Types	Measurement techniques	Subjective	Objective	Time of assessment	Dysregulation of post-chemotherapy cytokine levels	Association between cytokine level and cognitive changes
Gan 2011[51]	Prospective cohort	5 head and neck cancer	5 head and neck cancer	Cisplatin-based	IL-1β, IL-2, IL-4, IL-6, IL-8, IL-10, IL-12, TNF-α, IFN-γ, GM-CSF	Multiplex immunoassay	FACT-Cog	NP	T1: Pre-ctx;	X	X
									T2: mean 20m Post-ctx		
Ganz 2012[53]	Prospective cohort	49 breast cancer	44 breast cancer	Mixed regimens: Anthracycline-based, taxane-based	IL-6, IL-1RA, sTNF-RII, CRP	ELISA	Squire Memory Questionnaire	NP (and PET scan imaging)	T1: Post-ctx (Pre-endo tx);	√	√
									T2: 6 m;		
									T3: 12 m		
Janelsins, 2011 [55]	Prospective cohort (from a randomized clinical trial)	54 breast cancer	Not applicable	AC/CAF or CMF	MCP-1, IL-6, IL-8	ELISA	Cognitive items from Fatigue Symptom Checklist	Not applicable	T1: Cycle 2;	AC/CAF: √	AC/CAF: √
									T2: Cycle 4	CMF: X	CMF: X
Kesler 2012[41]	Cross- sectional	42 breast cancer (cytokines tested for 20 patients)	35 healthy (cytokines tested for 23 controls)	Mixed regimens: Anthracyline-based, 5-FU-based and MTX	IL-1β, IL-6, IL-8, IL-10, IL-12, TNF-α, IFN-γ	Multiplex immunoassay	Multifactorial Memory Questionnaire Ability Scale	NP (and hippocampal volume measurement)	Mean 4.8±3.4 years Post-ctx	√	√
Knobel, 2000 [50]	Cross-sectional	33 lymphoma	10 healthy	CHOP +/- methotrexate	IL-6, TNF-α, sTNF-p55 and p75 receptor	Immunoassay using cell-line cultures	EORTC-QLQ-C30- Cognitive functioning scale	N/A	Median 6 years from ctx	X	X
Meyers, 2005 [22]	Cohort	19 acute myelogenous leukemia;	Laboratory normative controls (for cytokine level assessment)	Lipadaunocin + cytoxan or topotecan, +/- thalidomide	IL-1β, IL-1RA, IL-6, IL-8, TNF-α	ELISA	Not applicable	NP	T1: Pre-ctx;	X	√ (association found between baseline cytokine level and cognitive status in the Ctx patients)
		35 myelodysplastic syndrome							T2: 1 m from T1		
Booth, 2006 [49]	Case-control	20 breast cancer	40 breast cancer	Mixed regimens: Not specified	IL-1β, IL-2, IL-4, IL-6, IL-8, IL-10, IL-12, TNF-α, IFN-γ, GM-CSF	Not specified	FACT-Cog	NP	Median 2 years post-diagnosis	X	X
Vardy, 2012 [45] a	Prospective cohort	176 colorectal cancer;	117 colorectal cancer;	Mixed regimens: oxaliplatin-based and irinotecan-based	IL-1β, IL-6, TNF-α, IFN-y, GM-CSF, IL-8, IL-10, IL-12	Multiplex immunoassay	FACT-Cog	CANTAB and NP	T1: Pre-ctx;	√	X
		72 metastatic colorectal cancer	72 healthy						T2: 6 m;		
									T3: 12 m		

Legend and abbreviations: (listed according to alphabetical order within categories)

a Additional data not shown; presented during oral presentation at the International Cognition and Cancer Task Force conference in 2012.

b AC: doxorubicin and cyclophosphamide; CAF: cyclophosphamide, doxorubicin and 5-fluorouracil; CMF: cyclophosphamide, methotrexate and 5-fluorouracil; CHOP: cyclophosphamide, doxorubicin, vincristine and prednisolone; MTX: methotrexate; 5-FU: 5- Fluorouracil.

c CRP: C-reactive protein; ELISA: enzyme-linked immunosorbent assay; GM-CSF: granulocyte macrophage colony-stimulating factor; IFN: Interferon; IL-: Interleukin-; IL-1RA: interleukin-1 receptor antagonist; MCP-1: monocyte chemotactic protein-1; sTNF-RII: soluble tumor necrosis factor receptor-II; TNF: tumor necrosis factor.

d CANTAB: Cambridge Neuropsychological Test Automated Battery; Ctx: chemotherapy treatment; Endo-tx: endocrine treatment; EORTC: European Organization for Research and Treatment of Cancer; FACT-Cog: Functional Assessment of Cancer Therapy – Cognitive Function; M: month; NP: traditional neuropsychological batteries; PET: positron emission tomography.

e “√” refers to the authors' observations that cytokine levels were elevated in chemotherapy-receiving study subjects, or the presence of a statistically significant correlation between chemotherapy and cognitive impairment; “X” refers to the authors' observations that cytokine levels were not elevated in chemotherapy-receiving study subjects, or the absence of a statistically significant correlation between chemotherapy and cognitive impairment.

The studies evaluated different cytokines, including pro-inflammatory cytokines, anti-inflammatory cytokines and cytokine receptors. The choice of cytokines for measurement was similar in most studies. All of the selected studies measured the serum levels of IL-1β, IL-6 or/and TNF-α. These cytokines were the biomarkers of interest in these studies probably because they have been shown to be strong candidates for mediating the components of sickness behavior[11], including cognitive impairment in neurological diseases such as Alzheimer's disease and multiple sclerosis[13], [30]. Baseline assessments of cytokine levels and cognitive functioning were performed in majority of the included studies. However, subsequent measurements were conducted at different time points, ranging from 24 hours to 3 years after chemotherapy administration.

Subjective and/or objective cognitive assessments were carried out to determine patients' cognitive status. Objective assessments involved traditional neuropsychological batteries such as Trail-making test A and B[56], the Hopkins verbal learning test[57], and computerized testing such as the CANTAB[58]. Subjective assessments included self-report questionnaires using the EORTC-QLQ-C30 health-related quality of life tool[59] and the Functional Assessment of Cancer Therapy-Cognition (FACT-Cog)[60]. Two of the studies used imaging techniques to evaluate the relationship among structural changes in the brain, cognitive impairment and cytokine dysregulation[41], [53].

### Summary of Selected Studies and Important Findings

Eight articles (six full manuscripts[22], [41], [50], [51], [53], [55] and two abstracts[45], [49]) were included in this review (Table 1). Information from full text and abstracts are presented separately.

One of the most recent studies was conducted by Ganz et.al[53]. This prospective cohort study involved 49 chemotherapy-receiving and 44 non-chemotherapy-receiving breast cancer patients. Assessment on patients' cognitive function was performed using an objective neuropsychological (NP) battery and self-reported Squire Memory Questionnaire, at baseline within 3 months of completing primary breast cancer treatment but before initiating breast cancer targeted endocrine therapy, 6 months later and 12 months later. Brain imaging with positron emission tomography (PET) scans was evaluated in a subset of patients. The authors observed an increase of sTNF-RII among chemotherapy exposed patients, as compared to non-chemotherapy receiving patients (2580±100 pg/ml vs. 2113±104 pg/ml; p = 0.0019). Higher baseline sTNF-RII in chemotherapy patients was significantly associated with increased self-reported memory complaints. In chemotherapy exposed patients, the longitudinal decline in sTNF-RII was significantly correlated with fewer memory complaints over 12 months (r = −0.34, p = 0.04). No signification association between objective neuropsychological tests and pro-inflammatory cytokines was reported.

In another recently published cross-sectional study, Kesler et.al[41] evaluated the association among serum cytokine levels, left hippocampal volumes and objective memory performance in 20 chemotherapy-receiving breast cancer patients and 23 healthy controls. The assessments were conducted 4.8±3.4 years post-chemotherapy. It was found that the breast cancer group had reduced left hippocampal volumes (p = 0.01) and cognitive performance and evaluated levels of IL-6 (p = 0.003) and TNF-α (p<0.0001), as compared to healthy controls. In the breast cancer group, in the breast cancer group, lower left hippocampal volume was associated with higher levels of TNF-α (β = −1.56; p = 0.04) and lower IL-6 (β = 0.734, p = 0.03). The interaction between TNF-α and IL-6 was also significant (β = 1.23; p = 0.04). These associations were not observed in the healthy control group. Authors concluded post-chemotherapy altered hippocampal volume and verbal memory difficulties may be mediated by TNF-α and IL-6 in breast cancer patients.

Janelsins et.al.[55] compared the severity of self-reported cognitive disturbances and cytokine dysregulation (IL-6, IL-8, and MCP-1) between 54 breast cancer patients who received doxorubicin-based (with cyclophosphamide, or cyclophosphamide plus fluorouracil; AC/CAF) chemotherapy or cyclophosphamide, methotrexate, and fluorouracil (CMF), prior to on-study chemotherapy cycle 2 and after two consecutive chemotherapy cycles. It was found that cytokine elevation of IL-6, IL-8, and MCP-1 was more apparent in patients who received doxorubicin-based group than the CMF group. Change in MCP-1 levels was negatively associated with self-reported forgetfulness (p = 0.019).

The final selected study that involved breast cancer patients was presented as an abstract[49]. Hence there is limited evidence and conclusion drawn from this abstract. This case-control study involved three groups of breast cancer patients: Cases who had received chemotherapy and self-reported cognitive dysfunction on the FACT-Cog questionnaire. The 2 groups of controls involved patients who received chemotherapy but did not report cognitive problems, and another group that had breast cancer but did not receive chemotherapy. Patients completed an objective NP, self-reported FACT-Cog and functional MRI. In this preliminary analysis, results showed that there was more pronounced self-reported cognitive impairment in chemotherapy-receiving patients than non-chemotherapy-receiving patients (p<0.0001). However, objective cognitive testing revealed that more cognitive impairment in non-chemotherapy-receiving patients than the other two groups. Increased activity in the frontal areas was observed activity in patients with greater objective cognitive impairment (p<0.0005). However, there was no significant correlation between symptoms and cytokine levels.

The other 4 studies were conducted in other cancer populations. In the study conducted by Gan et.al[51], cognitive functioning of five head and neck cancer patients who were treated with radiotherapy and another 5 cancer patients who received both with radiotherapy and cisplatin-based chemotherapy were evaluated using objective NP tests and self-reported FACT-Cog. They were assessed at baseline before their anti-cancer treatment and a mean duration of 20 months later. It was found that patients receiving cisplatin performed more poorly on objective CF than patients who received only radiotherapy. However, the difference was not statistically different and association was not established between objective cognitive functioning and patients' cytokine levels.

A total of 54 myelogenous leukemia or myelodysplastic syndrome were recruited in the Meyers et.al. study[22]. Objective NP tests were administered at baseline before the start of their chemotherapy treatment, and one month later. The patients' cognitive performances and cytokines levels were compared against population references and laboratory normative controls, respectively. At baseline, patients' cognitive functioning was below reference values. Higher IL-6 levels also were associated with worse performance on the executive functioning while higher IL-8 levels were related to better memory performance. However, it is important to note that, there were insufficient individuals that had follow-up cytokine levels and post-chemotherapy cognitive functioning. Hence, limited conclusions can be drawn from this study.

Knobel et.al.[50] evaluated fatigue severity in a cross-sectional study of 33 lymphoma patients 4–10 years after receiving the chemotherapy regimen ABMT. Patients also completed the self-reported cognitive functioning scale on the EORTC-QLQ-C30. Their results were compared to general population norms. Results showed that patients reported more cognitive disturbances than the reference population (mean EORTC-QLQ-C30 cognitive functioning score: 75 vs 89 points; p<0.001). Serum levels of IL-6, sTNFR-p55, and sTNFR-p75 were slightly elevated in all clinical groups of lymphoma patients compared to normal controls. However, the association between cytokine levels and cognitive disturbances was not assessed.

The last selected study is presented in an abstract[45]. This prospective study was conducted on colorectal cancer patients. The chemotherapy group consisted of 176 non-metastatic and 72 metastatic colorectal cancer patients, while the non-chemotherapy group consisted of 117 colorectal cancer patients and 72 healthy controls. Patients completed the self-reporting FACT-Cog, NP tests and CANTAB, a computerized NP battery at baseline (pre-chemotherapy), 6 months and 12 months later. Results showed that cytokine levels were elevated in the chemotherapy group at all 3 time points of assessments (Data not shown; presented during oral presentation at ICCTF Conference in 2012). However, there was no association between cytokine levels and cognitive functioning.

### Relationships among Chemotherapy, Cytokines and Cognition

Although some studies observed dysregulation of cytokine levels with chemotherapy[41], [53], [55], they reported conflicting evidence with regards to the strength and direction of the association between changes in cytokine levels and cognition. Ganz et.al. concluded that higher levels of sTNF-RII correlated with lower levels of baseline metabolism in the left inferior frontal gyrus in 17 breast cancer patients (r = −0.55; *p = 0.02*)[53]. The same study also found higher levels of sTNF-RII were significantly correlated with greater memory complaints on the Squire Memory Questionnaire after controlling for age, body mass index, radiation and depression, and time since last chemotherapy treatment among the 43 patients who received chemotherapy (r = −0.34, *p = 0.04*)[53]. Kesler et.al. observed that lower left hippocampal volume of the brain was associated with poorer verbal memory performance, higher levels of TNF-α and lower levels of IL-6 in 20 chemotherapy-receiving breast cancer patients[41].

Meyers et.al. observed that pre-chemotherapy levels of circulating IL-1, IL-6, IL-8 and TNF-α were higher in 54 chemotherapy-receiving cancer patients than in laboratory normal controls[22]. However, the same study did not assess the association between post-chemotherapy cytokine levels and cognitive changes.

The other studies did not find a significant correlation between cognitive impairment and cytokines[45], [49]–[51]. Notably, the conclusions drawn by some studies were limited by the small sample size[50], [51], or the lack of comparison with controls or baseline/follow-up assessments[22], [41], [50].

As chemotherapy agents are usually administered in combinations, it is more meaningful to evaluate the relationship between cytokine-induced cognitive impairment and chemotherapy regimens, rather than individual chemotherapeutic drugs. A recently published review also suggested that the accumulation of a combination of drugs, rather than individual drugs, may enhance neurotoxicity in cancer patients[4]. One study examined the effects of anthracycline-based (AC/CAF) chemotherapy and CMF on cytokine levels and cognitive impairment[55]. Significant increases in IL-6 (*p = 0.042*) and IL-8 (*p = 0.060*) levels were observed in the AC/CAF treatment group, whereas IL-6 was lowered in the CMF group (*p = 0.054*). Patients receiving AC/CAF experienced more severe symptoms including heavy-headedness, difficulty thinking, and problems with concentration. Similar degrees of forgetfulness were displayed in both groups of patients. Knobel et.al. stated that poorer cognition was observed among lymphoma patients who received a combination of cyclophosphamide, doxorubicin, vincristine, prednisone (CHOP) and methotrexate, compared with patients who received only CHOP (data not presented) [50]. This study provided preliminary evidence on how different chemotherapy regimens could affect cytokine levels and cognitive functioning differently. It was suggested that methotrexate's ability to cross the BBB and obstruct brain vascularization might have caused the cognitive impairment[61]–[63].

The time concordance between the onset of cytokine induction and the occurrence of cognitive impairment was not clearly elucidated in these studies. Notably, the current evidence could not ascertain the exact timing of the onset of cognitive impairment[64]–[67]. One study reported that the circulating level of sTNF-RII was elevated at 6 months post-chemotherapy and declined to a level similar to that of non-chemotherapy controls at 12 months post-chemotherapy and an increase in cognitive complaints from baseline was significantly correlated with increases in sTNF-RII levels (*p = 0.05*)[53]. Evidence on the onset and duration of cytokine induction thus remains inconclusive.

### Confounding Factors

Through this review, we identified a number of confounders that contributed to the heterogeneity of the studies which were not sufficiently accounted for. These confounders need to be evaluated and accounted for when studying the relationship between chemotherapy-associated cytokine induction and cognitive impairment. This discussion also forms the basis of our recommendations for future research.

1. **Effects of cancer.** The study populations differed among the selected studies; they included patients with different types and stages of cancers. Studies have shown that cancer cells are capable of producing cytokines [18], [29]. For example, it was found that TNF and transforming growth factor (TGF) were elevated in patients with early-stage cancers, whereas patients with progressive disease had elevated levels of angiogenin, granulocyte macrophage colony-stimulating factor and TNF-β [18]. Hence, it is difficult to determine whether an elevated cytokine level is partially or entirely caused by chemotherapy, the progression of cancer or a combination of both. Non-cancer controls may not fully eliminate the effects of cancer on cytokine levels as studies have shown that there may be a potential dysregulation of cytokine levels in subclinical cancer states observed in healthy subjects[68], [69]. Notably, individual variability (such as disease progression) can still exist within cancer controls. It is plausible that cytokine induction attributed by the different nature of cancers and anti-cancer treatments may cause varying levels of cognitive impairment in patients.
2. **Effects of gender.** Two studies evaluated the effects of gender on cognitive changes[46], [50]. Males demonstrated more cognitive impairment than females on objective cognitive tests, although subjective (self-reported) cognitive complaints were higher among females[46], [50]. It is postulated that higher levels of estrogen can offer neuro-protection, antioxidant properties[70] and maintenance of telomere lengths to protect against atrophic changes in the brain[71]. Although serum sex hormone levels have been measured[45], analysis was not performed to investigate their correlation with serum cytokine levels. The decrease in estrogen after menopause may increase pro-inflammatory IL-6 and TNF-α cytokine activity[24]. Higher subjective cognitive impairment among females may be attributable to neuroendocrine-related problems arising from chemotherapy-induced or aging-related menopause[72], [73]. Studies have also revealed that testosterone replacement, which can potentially shift the cytokine balance to a state of reduced inflammation and suppress the expression of the pro-inflammatory cytokines TNF-α, IL-1β and IL-6, has been shown to improve some aspects of cognitive ability in males[74]–[76]. Therefore, the receipt of anti-hormonal treatment must be taken in account in the assessment of cognitive changes in patients, especially in cancers such as prostate, testicular and breast cancers.
3. **Effects of aging.** The age of subjects across the selected studies ranged widely, from 18 to 84 years. With the exception of one study[53], age was not routinely adjusted as a confounding factor. It was proposed that cancer treatments can cause cognitive impairment through the acceleration of the aging process, and the biologic mechanisms underlying cancer, cancer treatments, aging, neurodegeneration, and cognitive decline are interconnected[77]. Aging may be associated with increase in IL-6, TNF-α and TNFR, regardless of comorbidities[30], [78]–[80]. Age-related impairment is also associated with increased hypothalamic IL-1β, which can damage the neuronal membrane through lipid peroxidation, leading to memory and learning deficits[12], [30]. Therefore, age is an important determinant in the measurement of cytokine levels and cognitive impairment[3], [81].
4. **Effects of genetic predisposition.** Most of the selected studies did not account for genetic predisposition. Only two studies performed genotyping for the apoE gene[45], [49], one of which reported that cognitive changes were not associated with the apoE genotype or elevated cytokine levels[45]. Yet, the apoE genotype has been associated with increased susceptibility to damage in the central nervous system and cognitive impairment[15]. Excessive secretion of pro-inflammatory cytokines such as IL-6 and IL-1 can occur in genetically predisposed individuals under stress[30], [82]. Genetic polymorphisms may influence cytokine activity in Alzheimer's disease and depression. It is worthwhile noting that other than the apoE genotype, polymorphisms in other cytokine gene variants may result in disrupted levels of cytokines[21], [26], [83] and may potentially affect cognition. One study discovered that a single-nucleotide polymorphism in the TNF-α-308 gene is associated with more severe cognitive deficits on three independent measures, namely, PAOFI Memory (*p* = 0.03), the Squire subjective memory questionnaire (*p* = 0.04) and the Multidimensional Fatigue Symptom Inventory – Mental (*p* = 0.005)[54]. Future study can focus on the identification of candidate genes that can influence cytokine activity and are associated with chemotherapy-induced neurotoxicity in cancer patients.
5. **Effects of disturbances in the sleep-wake cycle.** Increased serum cytokine levels have been found among cancer patients with dampened circadian rhythms[27]. Disordered sleep induces an inflammatory response that leads to an increase in IL-6 and TGF-α. These cytokines, termed nocturnal cytokines, have been shown to affect the sleep-wake cycle, causing a disruption in the neuroendocrine control of cortisol release, resulting in further release of pro-inflammatory cytokines[23]. Moreover, dysregulation in sleep is linked to cognitive impairment in the domains of attention, executive function and motor control ability[30], [84]–[86].
6. **Effects of psychosocial characteristics (fatigue, depression, anxiety and stress).** Fatigue, depression, anxiety, distress and mood changes have been found to reduce patients' cognitive functioning and could be mediated by chemotherapy-induced cytokines. A meta-analysis revealed a correlation between fatigue and serum cytokine levels of IL-6 and IL-1RA[87]. However, none of the three selected studies[45], [49], [50] that measured fatigue as one of their endpoints showed a correlation between cytokine levels and the occurrence of fatigue. Depression is found to disrupt the hypothalamic-pituitary-adrenal axis and production of cortisol and pro-inflammatory cytokines[13], [29], [30], [88], [89] through a positive feedback mechanism. To reinforce this relationship, studies have shown the effects of antidepressants on the reduction of pro-inflammatory cytokine levels[19]. Note that patients' reports of cognitive problems during chemotherapy treatment were typically not related to objective cognitive test results, but to psychosocial parameters such as anxiety and depression[90].

### Directions for Future Research

Based on the evidence and limitations of this review, a list of recommendations is proposed to improve the investigation of chemotherapy-induced cytokines as a biological determinant of cognitive changes in cancer patients (Table 2). To summarize, in line with ICCTF's recommendations, it is suggested that studies of this subject matter should include a longitudinal design, appropriate choices of chemotherapy-receiving cancer patients, disease specific and healthy controls, inclusion of baseline and post-chemotherapy standard neuropsychological assessments and robust statistical analysis of longitudinal data[91]. In addition to these recommendations, it is also important to standardize the protocol and timing for the blood draw and measurement of cytokine levels, and account for the confounding factors mentioned in Figure 1. In particular, studies should control for anxiety, depressive and fatigue symptoms that are known widely in literature to be associated with cancer- and chemotherapy-induced cytokine activity[29].

**Table 2:** Recommended methodological guidelines for future studies.

Criteria	Description	Checklist
	Experimental methods	
Inclusion of suitable controls (non-chemotherapy cancer patients with similar demographics).	To account for cytokine levels and cognitive impairment in cancer patients without chemotherapy.	Did the study include controls who do not require chemotherapy, and who had the identical type of cancer and demographics as the sample population?
Account for co-morbidities	To account for changes in cytokine levels and cognitive impairment as a result of chemotherapy, rather than other factors.	Did the study account for non-cancer disease states?
(anemia, depression, anxiety, Alzheimer's disease, multiple sclerosis, Parkinson's, dementia, traumatic brain injury, etc.).		
Account for other forms of treatment than chemotherapy.	To account for changes in cytokine levels and cognitive impairment as a result of chemotherapy, rather than other forms of treatment.	Did the study account for patients who were receiving other forms of therapy that may affect cytokine levels and cognitive impairment?
(hormonal therapy, cytokine therapy, radiation, antidepressants, etc.).		
	Chemotherapy information	
Inclusion of specific chemotherapeutic agents or regimens for assessment.	To understand the pharmacological effects of cytokine-induced cognitive impairment.	Did the study provide clear data regarding the type of chemotherapeutic drugs/regimen involved, the duration, cycles, doses, dose intensity and route of administration of the chemotherapy treatment?
Inclusion of specific dose intensity, route of administration and duration of chemotherapeutic treatment.	To understand whether there is a correlation between route of administration/strength and duration of dosing with cytokine levels and cognitive impairment.	
	Cytokine measurement	
Inclusion of both pre-chemotherapy baseline and post-chemotherapy assessments at appropriate intervals.	To account for changes in cytokine levels as a result of chemotherapy by comparing baseline measurement with post-chemotherapy measurement.	Were measurements carried out before and after patients received chemotherapy/surgery?
If applicable, inclusion of pre-surgical baseline and post-surgical assessments at appropriate intervals.	To account for changes in cytokine levels as a result of tumor progression/tumor load by comparing pre-surgical measurement with post-surgical measurement.	
Inclusion of specific cytokines involved in the study.	To identify the specific cytokine and its effects on cognitive impairment.	Did the study specify the types of cytokines to be tested for?
Accounting for accuracy of testing.	To minimize discrepancies in results due to the handling and processing of samples.	Did the study utilize published procedures for the testing of cytokine levels?
Inclusion of standardized timing across the samples for extraction of blood samples.	Different timings of extraction may result in different levels of cytokines.	Did the study indicate a specific time for the extraction of cytokines from the sample and control population?

To evaluate the role of cytokines as mediator between chemotherapy and cognitive impairment, it is recommended that investigators could provide a rationale behind the choice of cytokines or biomarkers tested in the studies. Other than the known pro-inflammatory cytokines (such as IL-1β, TNF-α and IL-6) that are associated with cognitive impairment, it may be worthwhile to evaluate the relationship between neurotoxicity and other less explored biomarkers, such as anti-inflammatory cytokines and vascular endothelial growth factor (VEGF) which may potentially exert a neuro-protective effect. For example, it has been suggested that VEGF can stimulate angiogenesis and modulate vascular permeability and promote neurogenesis[92]. Novel technologies such as multiplex immunoassay can be applied to enable the analysis of several cytokines of interest simultaneously[45], [93]. Commercially available bead-conjugated antibodies permit the measurement of up to 25 different cytokines within a sample[93]. Validation studies have shown that results of multiplex immunoassay well correlate with traditional enzyme linked immunosorbent assay (ELISA) for a large majority of human cytokines and biomarkers[94], [95]. Multiplex immunoassay also requires lower-volume samples to detect cytokines over a broad dynamic range of concentrations in a more cost-effective way[93], [96]–[98]. This advantage is favorable for longitudinal studies that have high attrition rates, as there may be a limited number of samples to conduct ELISA[93]. If resources allow, key genotypes that affect cytokine-induced cognitive impairment should be explored to investigate the role of genetic determinants in the induction of cytokines.

The multi-factorial characteristic of this type of study also calls for a more robust analysis of the statistical data and an account of the various etiological agents involved. The use of Principal Component Analysis (PCA)[99] is suggested to facilitate the reorganization of data and examine the causes of variance in the data. For example, one group successfully used this technique to identify the association between baseline cancer-related fatigue and IL-6, IL-6R and IL-17 (variance  =  78%)[100].

Other than clinical studies, animal models also offer several advantages for evaluating the effect of chemotherapy on cognitive outcomes[101]–[104]. Animal models can eliminate numerous confounders outlined in this review, such as the cancer itself, psychosocial characteristics and presence of other comorbidities. The time of administration of chemotherapeutic drugs, behavioral assessments and blood draw can be standardized in animal studies to reduce the variability observed in clinical studies. Results from animal models can aid in elucidating the biological mechanisms involved in chemotherapy-induced cognitive changes and the specific underlying brain regions involved.

All current studies of this subject matter attempt to measure cytokine concentration in patients' serum/plasma, and not the cerebrospinal fluid (CSF). However, it is logistically challenging to obtain CSF from subjects in a research setting. It is proposed that for future studies that involve intrathecal administration of chemotherapeutic drugs, especially in leukemia patients, CSF can be collected to examine the relationship between the concentration of cytokine in CSF and cognitive functioning before and after the cancer treatment.

Results from recent published imaging studies also support the hypothesis that chemotherapy affects the brain structure and provide research directions for candidate mechanisms of chemotherapy-associated cognitive change. Two included studies examined the relationship among structural changes in the brain, cytokine levels and cognitive impairment[41], [53]. One of the studies found that IL-6 and TNF-α levels and hippocampal volumes were associated with verbal memory functioning despite its relatively small sample size[41]. Imaging studies play an important role in complementing and providing support for the role of pro-inflammatory cytokines in chemotherapy-associated brain injury and subsequent cognitive impairment. It allows researchers to identify the specific site of cognitive impairment and their relationship with physiological biomarkers.

Finally, research gaps remain to be filled regarding the role of cytokines in relation to cognitive changes in cancer patients (Table 3). Other than understanding the pattern of changes in cytokine levels during cancer treatment, it is also important to elucidate the effect size of the change that constitutes a significant clinical difference in cognitive functioning. Striving towards the answers to these research questions will lead to a better understanding of this neurocognitive phenomenon in cancer patients. It would be worthwhile to reflect upon what we know currently about this subject matter, and determine whether these terms are appropriate in describing this phenomenon. The terms “chemobrain” or “chemofog” may not accurately reflect the complexity of the multiple potential etiologies contributing to this problem[105]. Results of this review suggest that cognitive impairment may represent a component of the cytokines- and inflammatory markers- mediated cluster of cancer- and treatment- related symptoms, such as fatigue, depression and stress.

**Table 3:** Summary of potential research questions.

What are the specific chemotherapy-induced cytokines that are associated with cognitive changes? How strong are these associations?
What are the specific chemotherapy-induced cytokines that are not associated with cognitive changes?
Do particular chemotherapy regimens and/or drugs affect the types and extent of changes in cytokine levels?
Are the changes in cytokine levels dose-dependent?
What is the amount of change in cytokine levels that constitutes a clinical change in cognitive function?
In epidemiological terms, are cytokines alone “necessary-and-sufficient” to cause chemotherapy-induced cognitive changes?
Does genetic variation lead to changes in chemotherapy-induced cytokine levels and severity of cognitive changes?
What is the relationship between the dysregulation of cytokine levels, structural brain damage and cognitive impairment?
Do cytokine levels return to normal levels after the cessation of chemotherapy?

### Limitations of this review

Despite the well-defined inclusion criteria, the reviewed articles were heterogeneous in nature and a quality appraisal strategy could not be applied to them. Knowledge on the subject matter is in its infancy, and the availability of literature to substantiate and justify the findings from the selected studies is sparse. Moreover, conclusions drawn from the two abstracts are tentative in nature and subjected to changes when they are published.

## Conclusion

Although some of the selected studies observed that patients on chemotherapy experienced cognitive impairment and dysregulation in pro-inflammatory cytokines of IL-1β, IL-6 and TNF-α, there is limited evidence to suggest that there is an association between the severity of cognitive impairment and cytokine dysregulation. Our results suggest that the intermediary role of cytokines in post-chemotherapy cognitive impairment is still controversial and requires further evaluation. Notably, due to the high heterogeneity of the studies included in this review, it is difficult to draw conclusive inferences regarding the causal relationship between chemotherapy and cytokine-induced cognitive impairment.

Understanding the mechanisms of the cytokines as mediators in chemotherapy-induced cognitive impairment will improve our knowledge of the clinical implications of ‘chemobrain.’ It is anticipated that the harmonization of methodologies will allow the pooling of results from robust studies and thus provide further insights on this subject matter. Such information will enable scientists with pharmacological insights to formulate therapies or appropriately adjust the current treatment regimes to minimize the effects of cytokine-induced cognitive impairment in patients with cancer.

## Supporting Information

Checklist S1 **PRISMA 2009 Checklist**
(DOCX)
